# Risk Field of Rock Instability Using Microseismic Monitoring Data in Deep Mining

**DOI:** 10.3390/s23031300

**Published:** 2023-01-23

**Authors:** Longjun Dong, Huanyu Zhu, Fang Yan, Shuijin Bi

**Affiliations:** School of Resources and Safety Engineering, Central South University, Changsha 410083, China

**Keywords:** risk assessment, microseismic monitoring, severity, probability, risk field

## Abstract

With the gradual depletion of surface resources, rock instability caused by deep high stress and mining disturbance seriously affects safe mining. To create effective risk management, a rock instability risk field model using microseismic monitoring data is proposed in this study. Rock instability risk was presented visually in 3D visualization. The in-situ microseismic monitoring data was collected and analyzed to make calculation of peak ground velocity (PGV), peak ground acceleration (PGA), energy flux, energy and seismic moment. Indicator weights of PGV, PGA, energy flux are confirmed by using the analytic hierarchy process (AHP) to calculate risk severity. The Copula function is then used to solve the joint probability distribution function of energy and seismic moment. Then the spatial distribution characteristics of risk can be obtained by data fitting. Subsequently, the three-dimensional (3D) risk field model was established. Meanwhile, the established risk field is verified by comparing monitoring data without disturbance and the blasting data with disturbance. It is suggested that the proposed risk field method could evaluate the regional risk of rock instability reasonably and accurately, which lays a theoretical foundation for the risk prediction and management of rock instability in deep mining.

## 1. Introduction

Mineral resources are not only one of the important pillars for the survival and development of human society but they are also the material guarantee for national economic and social development. However, with the increase in mining depth comeshigh risk. Mining disasters caused by rock instability become more prominent in deep mining and this consequently reduces production safety and economic benefits of mines [1]. Acoustic emission technology is of great significance to rock strength monitoring, instability assessment and precursor warning [2]. In addition, it is of paramount importance in analyzing the characteristics of rock instability precursors and the direction of principal stress which are critical for the prevention of geological disasters. It is also the theoretical basis of in-situ microseismic monitoring technology [3]. The stress, strain, displacement and microseismic activity of the mined rock instability could be obtained in real time by the in-situ monitoring data. This in-situ monitoring data is helpful in improving the risk management in deep mining. Therefore, the study of microseismic risk assessment is a good prospect for the improvement and development of risk analysis and management in deep mining basing on in-situ monitoring data.

The microseismic monitoring technique is important in carrying out geo-stress monitoring in deep mining. Its principle is to reflect the accurate condition of rock instability by analyzing the mechanical parameters contained in microseismic events [4]. Therefore, the accurate pickup and discrimination of microseismic events and explosion signals determine its timeliness and accuracy. Ma et al. used full waveform inversion and statistical methods to discriminate the type of induced seismicity in deep mining [5]. Dong et al. proposed discriminators which have explicit and simple functions based on the three statistical techniques: the Fisher classifier, naive Bayesian classifier and logistic regression [6]. These methods provide important reference for picking up and distinguishing microseismic events. During the analysis process of the in-situ monitoring data, accurate calculation of the potential location of rock instability is important for subsequent data processing. Ma et al. used multi-point synchronous data acquisition to define microseismic source position and arrival time [7]. Dong et al. used the velocity free MS/AE source location method to study the variation of MS/AE location accuracy and spatial evolution characteristics of granite faults under complex stress conditions [8]. The abnormal arrivals with different scales of errors can be recorded by the monitoring sensors, which usually leads to large errors between the located results and the authentic coordinates. Wang et al. combined the wavelet packet analysis and cross-correlation technology to obtain more accurate AE signal delay to reduce the positioning error [9]. Dong et al. proposed a collaborative localization method using analytical and iterative solutions (CLMAI), which combined with the arrivals of multi-sensor and inversion of the real-time average wave velocity, to seek the optimal locating results [10]. The proposed method is a beneficial complement for the current iterative methods using premeasured velocity. This method is applicable to microseismic sources localization under complex abnormal arrivals in the rock mass structure of dynamic underground mining. However, with the continuous development and optimization of travel time tomography identification technology, a new method proposed by Dong et al. can also be applied to define the potentially risky area [11]. The development of the real-time monitoring has conversely made the detection of abnormal regions in complex structures a challenging target. Hlousek et al. used wide-angle measurement data to reach tomography and obtained the three-dimensional (3D) velocity model of depth imaging [12]. Dong et al. proposed an improved three-dimensional (3D) tomography method combining passive acoustic emission acquisition and active ultrasonic measurements [13]. The method is important not only for the identification of potentially hazardous areas but also for the improvement and optimization of positioning accuracy. Since the rock fracture characteristics and principal stress directions are important for prevention of geological disasters, Li et al. found that the direction of intermediate principal stress plays an important role in inducing the direction of rock crack propagation [14]. Zhao et al. used microseismic monitoring and borehole imaging to observe rock fracture and clarify the mechanism of rock mass mechanical behavior [15]. Dong et al. considered the acoustic emission characteristics and anisotropy of wave velocity variation of granite under two-dimensional stress in combination with the acoustic emission (AE) technique [16]. Additionally, because microseismic monitoring technique is important for mining risk assessment, Huang et al. used it to propose an effective evaluation method of water inrush from coal mine floor [17]. Gai et al. went on to use microseismic monitoring and ArcGIS technology to study the dynamic load disaster of coal mines in order to draw the risk level zoning map [18]. Ma et al. used microseismic monitoring technique and moment tensor inversion to define the fault types of key strata, which provided theoretical reference for mining risk assessment [19]. Microseismic monitoring is also applied in the safety evaluation research of tailings dam. Dong et al. proposed some developments and new insights for environmental sustainability and disaster control of tailings dam, which also included the principle of regional risk assessment [20]. He et al. combined the electrical emission (EME) and micro-seismic (MS) monitoring to establish the coupling evaluation system based on the monitoring results of specific rock bursts in coal mines [21]. Liu et al. used microseismic monitoring technology to evaluate the rock stability of Hongtoushan Copper Mine [22]. All these studies reflect the wide application of microseismic monitoring technology in the field of mining risk assessment. However, the relationship between risk and coordinates in the spatial monitoring area was not established in all these studies. Thus, in order to describe the distribution of risk in space more intuitively and effectively, the three-dimensional (3D) risk field theory has been put forward and developed rapidly.

Risk assessment can be divided into 2D risk assessment and 3D risk assessment according to the spatial dimension of the calculated variables. 2D risk assessment which can also be considered as regional risk assessment or risk mapping has been widely used in the early risk warning of chemical industry clusters [23], the mapping of risk distribution for arsenic-contaminated land [24], regional risk ranking of environmental chemical stressors [25] and other fields [26]. Gai et al. provided an assessment framework for regional evacuation of major incidents [27]. For flood risk assessment, Christie et al. proposed a Coastal Risk Assessment Framework (CRAF) to determine the greatest flood risk in flood risk areas [28]. Wang et al. conducted a regional flood risk assessment in the Huaihe River Basin in China and the flood risk was simulated by a two-dimensional hydrodynamic model [29]. For research fields of resource exploration and management, Dong et al. proposed a new method for empty region identification in the two-dimensional complex structure [30]. Yan et al. proposed a novel hazard assessment method called extended set pair analysis (ESPA) based on set pair analysis (SPA) [31]. However, with the continuous deepening of research, some researchers have found that 2D risk assessment cannot be applied well to research under complex sample conditions. Smerzini and Pitilakis proposed numerically computational 3D seismic risk assessment modeling based on physics [32]. Szoke et al. [33] simulated developments of real-time 3D radiation risk assessment. The authors built a mathematical model to calculate radiation-related parameters and the 3D radiation risk distribution was obtained through computer simulations. Suddle and Ale [34] indicated that t height is a crucial parameter for the 3D risk assessment. Chemical facilities and instruments can also be analyzed better by 3D risk assessment [35]. This gradual growth and improvement of the theoretical system of 3D risk assessment, has resulted in it being applied in the petroleum and chemical industry fields. In Grassi’s study, an improved 3D risk assessment method was presented for assessing the vulnerability of satellites to space debris. The results of the study showed a significant reduction of vulnerability [36] and this inspired the application of 3D risk assessment in many fields. Thus 3D risk assessment can also be applied to rock instability using microseismic monitoring data in deep mining.

With the development of microseismic monitoring technique, more accurate data can be collected by arranging a certain number of sensors at suitable locations. Thus, the data samples are statistically analyzed so that the probability and severity of risk occurrence are quantitatively characterized and the quantitative model of risk area characteristics can be established on the entire monitoring site area. The relationship of risk and coordinate in the monitoring area will then be established. Therefore, the model can reveal the quantitative characterization of the risk value of the assessed area to achieve effective risk prediction.

## 2. Materials and Methods

### 2.1. Risk Field

Based on the three-dimension risk assessment principle of field theory, if the risk value of any point in the spatial assessment area can be calculated, then a three-dimensional risk field model can be established [37]. The parameters such as magnitude, peak ground velocity (PGV), peak ground acceleration (PGA), energy, etc., should be obtained in the monitored area. Under normal conditions, they should be within the normal range when no abnormality occurs thus the risk level in the monitoring area under these conditions should be at a normal acceptable level.

We could assume that a certain abnormal state occurs in the monitoring area with the introduction of certain characteristics. Depending on the actual situation, some physical parameters in the area can have abnormal changes such as the energy, magnitude, seismic moment, PGV, PGA and so on. These parameters can reflect the changes in the physical quantity of the monitoring area to a certain extent. Basing on the above risk field theory, we can therefore analyze the risk value from the perspective of severity and the probability. Microseismic monitoring technique can be used to locate the epicenter position through the in-situ arranged sensors so that the related parameters can be calculated [38]. If the coordinates of the sensor monitoring point are known then the following calculation can be performed using severity and probability and the risk value at the monitoring point can be obtained.

**Figure 1:**
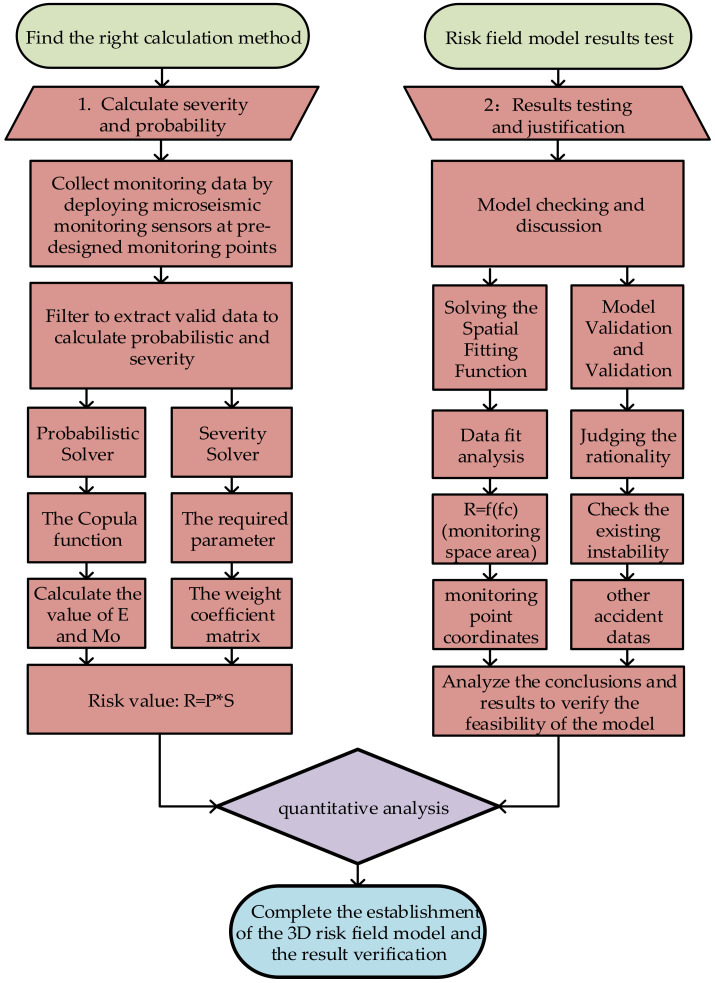
Flow chart showing how toestablish spatial 3D risk field prediction model based on monitoring data.

The relationship between the risk value and the monitoring point coordinates can be established in this way, the risk of each sensor can be obtained by arranging the microseismic monitoring data and the risk value of the monitoring area can be obtained by fitting and analyzing the data collected by each sensor. Hence, the relationship between the risk in the monitoring area and the spatial coordinates can be established. Figure 1 shows the flow chart of the application of the risk field theory to the in-situ microseismic monitoring data so as to obtain the risk associated with the above analysis.

### 2.2. Probability

The monitoring data obtained by effectively triggered sensors is extracted from the in-situ microseismic monitoring sensors. The accuracy of the calculation results is determined by the number of sensors effectively triggered. 28 sensors were arranged in the monitoring process in total and the microseismic monitoring data with more than 10 effectively triggered sensors was selected for calculation.

Parameters that can effectively reflect the characteristics of the monitoring area to achieve the quantitative characterization of monitoring data should be selected. Energy and the seismic moment are selected as the parameters to calculate the risk probability value of the monitoring area in this study.

Since the joint probability distribution function can be solved by the Copula function, we can select the data sample and draw the frequency distribution histogram of energy and seismic moment. The Copula function is an effective method to construct the joint distribution function of related non-normal variables [39].

The joint distribution function expression of two-dimensional parameters is used in this paper is F (E, Mo).

The corresponding joint probability density function expression is f (E, Mo)

According to Sklar’s theorem, the joint distribution function is [40]:

F (E, Mo) = C (E, Mo; θ) = C (μ_1_, μ_2_; θ) (1)

The joint probability density function is:

f (E, Mo) = D (E, Mo; θ) f_1_ (E) f_2_ (Mo) (2)

In the formula, the physical meaning of each parameter is as follows:

μ_1_ = F_1_ (E), μ_2_ = F_2_ (Mo) are the one-dimensional distribution functions of each parameter, respectively.

f_1_ (E), f_2_ (Mo) are the one-dimensional probability density functions of the corresponding functions, respectively.

C (μ_1_, μ_2_; θ) is the two-dimensional Copula function.

D (E, Mo; θ) is the two-dimensional Copula density function.

### 2.3. Evaluation of Severity

Various characteristic levels of the monitoring area can be intuitively reflected by parameters such as PGV, PGA, magnitude and so on. Since there are many influencing factors, it is not practical to use some representative as the solution criterion for severity only from the perspective of a single variable. The method of qualitative and quantitative analysis on the basis of this condition is therefore adopted in this study and the quantitative characterization of the severity can then be obtained. PGV, PGA, and energy flux are selected as the parameters for solving the severity in this study. Since the purpose of this study is to set a risk field prediction model, it is necessary to explain the severity judgment standard which is mainly divided into two steps. The first step is dimensionless parameterization and another one is to establish the weight coefficient matrix [41].

The dimensionless processing method is used to determine the critical maximum value PGV_max_, PGA_max_, and E(f)_max_ of the monitoring value of this parameter in the microseismic monitoring data within a certain period of time, then set S_1_ = PGA/PGA_max_, S_2_ = PGV/PGV_max_, S_3_ = E(f)/E(f)_max_ (PGV, PGA, E(f) are in-situ monitoring values).

**Figure 2:**
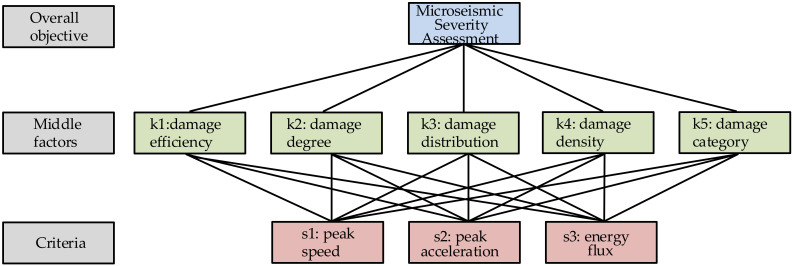
Theoretical framework for establishing weights by AHP.

Figure 2 shows the theoretical framework for establishing weights by AHP and Table 1 shows the weight distribution for each indicator.

When the weights are well established, the severity S is then calculated as follows:

α = [ϑ_1_, ϑ_2_, ϑ_3_], β = [S_1_, S_2_, S_3_], S = α × β^T^ (3)

### 2.4. Risk Field Prediction Model

In order to obtain the rock instability risk field model in a certain space region, the data collected by the sensors is selected to calculate the parameters required for the calculation. The risk of the coordinates of any point in the space region can then be obtained by the above analysis [42]. The following derivations can also be obtained by the above analysis. We can make conditional assumptions using the following steps: Firstly, we need to locate the source coordinates for the microseismic monitoring event. The risk can then be set as a function since the required parameters are all related to the distance to the epicenter. However, due to the complex and changeable field conditions, only the ideal situation is considered in this study and the external interference factors that may be caused by a series of other reasons are excluded. Finally, the fitting solution will be carried out.

Based on the above analysis for a microseismic monitoring event, we could set a certain point fc (x, y, z) in the monitoring area as the distance to the epicenter:

(4) $$X=\sqrt{{{(x\;-\;x}_{0})}^{2}{+(y\;-\;y}_{0}{)}^{2}{+(z\;-\;z}_{0}{)}^{2}}$$

Combined with the above calculation: the risk value at point fc (x, y, z) has the following calculation formula:

R (fc) = P (fc) S (fc)(5)

In this formula, P (fc) is calculated as follows:

P (fc) = P [f_1_(X), f_2_(X)] (6)

P [f_1_(x), f_2_(x)] is a two-dimensional Copula function with parameters E and Mo.

S (fc) is calculated as follows:

S (fc) = ϑ_1_ f_3_ (X) + ϑ_2_ f_4_ (X) + ϑ_3_ f_5_ (X) (7)

If the equation of each parameter in relation to the source distance X can be given explicitly, f_1_(X), f_2_ (X), f_3_ (X), f_4_ (X), f_5_ (X) are the functions of the parameters E, Mo, PGV, PGA, and E(f) respectively. However, after considering many factors such as calculation errors, positioning accuracy, small amount of data and discontinuous data recording, the study focuses on the risk field model theory for a single microseismic event. Based on the above analysis, f_1_(X), f_2_ (X), f_3_ (X), f_4_ (X), f_5_ (X) are the discrete data of each parameter corresponding to a single microseismic event record.

ϑ_1_, ϑ_2_, ϑ_3_ are the weights of parameters PGV, PGA, and E(f) respectively.

Finally, the formula for calculating the value at risk is obtained:

R (fc) = P (fc) S (fc) = P [f_1_(X), f_2_(X)] [ϑ_1_ f_3_ (X) + ϑ_2_ f_4_ (X) + ϑ_3_ f_5_ (X)] = ϑ_1_ f_3_ (X) P [f_1_(X), f_2_(X)] + ϑ_2_ f_4_ (X) P [f_1_(X), f_2_(X)] + ϑ_3_ f_5_ (X) P [f_1_(X), f_2_(X)] (8)

## 3. Results

### 3.1. Case Study

The monitoring data of a phosphate mine in a certain area in southwest China is used for a certain period of time in this study. The location of the collection point is determined by the actual needs of the site and microseismic sensors are deployed according to the site requirements to monitor the situation in real time. The analysis of microseismic monitoring data mainly follows the following steps: Firstly, microseismic monitoring data in the corresponding monitoring area can be obtained through the monitoring stations. The basic principle to improve the data accuracy is noise reduction [43]. The data can then be collected by each station among the data exchange center and summarized to the ground microseismic server.

Usually the data is divided into surface monitoring and underground monitoring during the actual microseismic data collection process. Surface data monitoring is usually relatively simple and the stations can be directly arranged in the corresponding monitoring area for direct monitoring [44]. Depending on the surface conditions, data collection and transmission is difficult and the data exchange center is usually required to summarize the collected data. The risk can be calculated together with the data value recorded by the sensors in order to ensure that the collected data will not be lost as much as possible and to safely transmit the data to the surface service center effectively.

### 3.2. Severity Calculation

Based on the analysis in Section 2.3, we selected the in-situ monitoring data at the time of t0: 201401040322. Table 2 shows the calculation results of severity.

### 3.3. Probability Calculation

**Figure 3:**
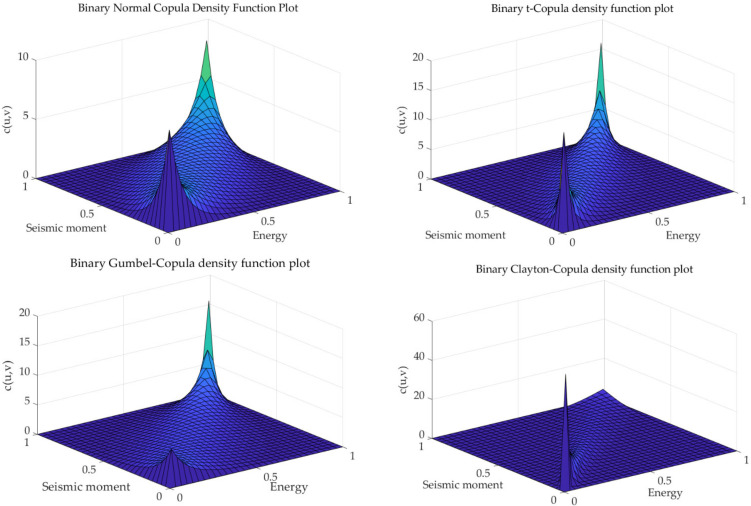

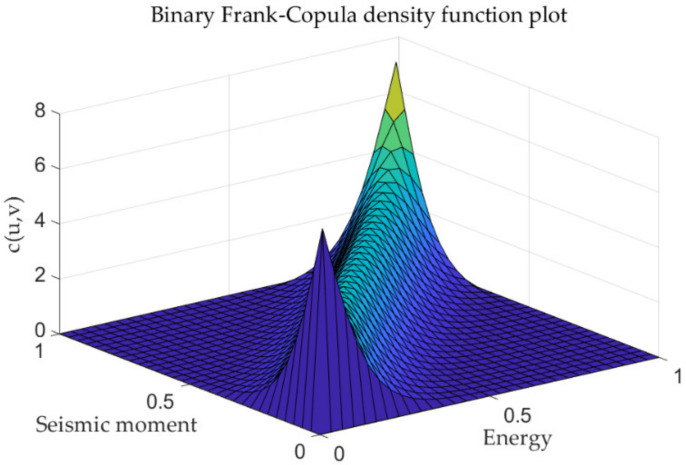
Probability density map of common Copula functions.

**Figure 4:**
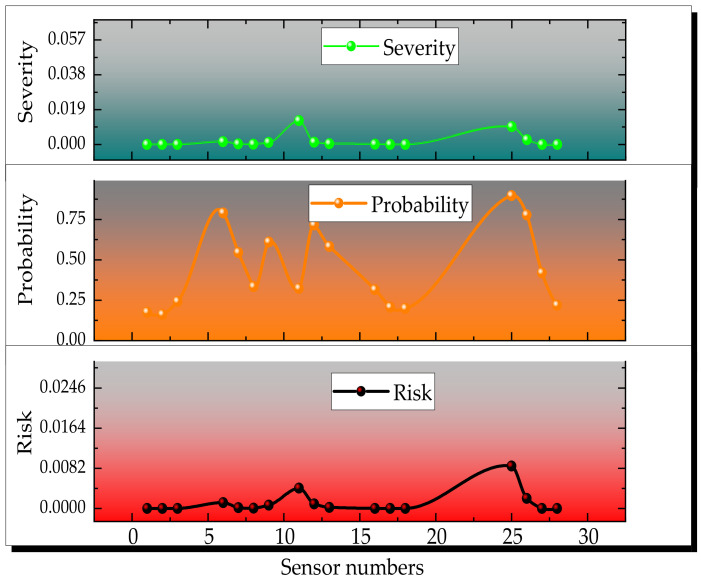
Calculation results of the risk value of sensors.

Figure 3 shows the probability density map of common Copula Functions. Different functions describe the correlation between variables, their associations and the differ encein their correlation structures. The AIC criterion and the BIC criterion are frequently used to quantitatively judge the fitting ability of different functions. In applying these two identification criteria for optimal function identification, the Copula function with the minimum AIC value or BIC value is considered to be the optimal function for fitting the correlation structure of the original observations [45]. Table 3 shows the AIC and BIC value calculation results. Figure 4 shows the risk value of the calculated results in Table 4.

### 3.4. Rock Instability Risk Field

During data processing, the optimal fitting function model is selected in combination with the selected analysis data. The optimal function model can then be selected to fit and analyze the data until convergence. If the fitting results do not converge, then reselect the model until convergence is successful. The optimal fitting curve function model is obtained by analyzing the residual error, fitting degree, and other error statistical analysis.

Using the above analysis, combined with the calculated risk value results and the known sensor and source coordinates we can consider that the risk value of the sensor nearest to the source location is the largest in this study. This is an important basis for screening the calculated results. However, in view of the complexity and error of the in-situ environment, in-depth research will be carried out to optimize the screening conditions in the future. This study mainly considers only the risk distribution under ideal conditions. Consequently, a three-dimensional scatter diagram corresponding to the three-dimensional coordinates of the sensor and the risk value can be drawn in space.

After selecting the data within a certain range, we can assume that the actual effective detection distance of the sensor in the actual monitoring process is within the range of 1000 m and the source distance is within the approximate range of 0–1000 m. Table 4 shows the magnitude of the risk value. We can get the attenuation trend of its distance from the source by fitting results so that the change of the risk value in each risk area can be reflected by them.

**Figure 5:**
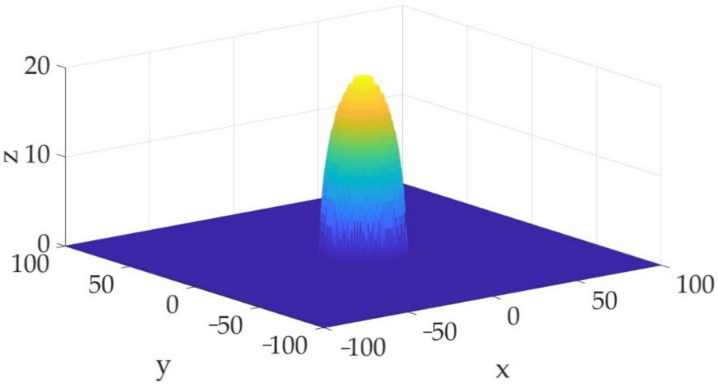
The iso-surface with a risk value of 10^−3^ at the moment of t0: 201401040322.

The attenuation trend can be analyzed and obtained when the source distance is equal to a certain value in the interval, which represents the distance of the propagation of the risk value [46]. Figure 5 shows the monitoring data at time t0: 201401040322 and this is selected to establish a three-dimensional risk field model in a local area combined with the above analysis.

**Figure 6:**
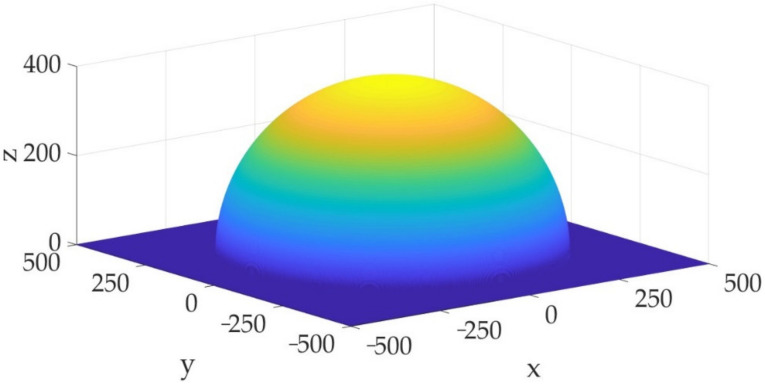
The iso-surface with a risk value of 10^−4^ at the moment of t0: 201401040322.

Figure 6 shows the other set of monitoring data selected for analysis to establish another three-dimensional risk field model combined with the above analysis.

## 4. Discussion

### 4.1. Verifying the Validity and Availability

In order to verify the accuracy of the model, the reliability of the model is checked by comparing the instability data existing in the field. If a clear comparison can be made, an objective comparison result can be reflected. Furthermore, it can be proved that this method can effectively reflect the changing laws of risk trends in this field. In this study, we selected the monitoring data without disturbance and the monitoring data with disturbance occurred for comparison and verification.

The selected data with disturbance for testing is T1: 201401040557, T2: 201401130504, and T3: 201401210347. Since the recorded data has time intervals, the corresponding monitoring data without disturbance is: t1: 201401040529, t2: 201401130537, and t3: 201401210428. Table 5 shows the calculation results compared with the above analysis.

Three groups of microseismic monitoring data are selected for this experimental comparison. They are mainly selected based on the combination of the recorded data included, equipment performance, positioning accuracy and other factors. The time interval selected for the comparison data is within 10 s to ensure continuity. The number of sensors triggered under normal monitoring state is different from that under the unstable disturbance state. Also the number of sensors triggered under the unstable disturbance state is usually large because of the large vibration amplitude. Some sensors that are obviously triggered in the unstable state are not triggered in the normal state. Sensors that trigger data are obviously selected for comparison in this study. Since the original waveform recorded by the experimental equipment needs to be calculated by the algorithm, the results recorded will be affected by the positioning accuracy. Although positioning accuracy is the main cause of error, basing on the above analysis and prediction, with the gradual optimization of the positioning algorithm, the data error value will become smaller and the accuracy as well as the practicality of the model will be improved. From the above table, the analysis of all data results in the three sets of comparative data can be known. The prediction results of the No. 18 sensor in the first group and the second group have deviations but the other groups of data all meet the expectations. Furthermore, the calculated risk value without disturbance is smaller than the risk value with disturbance at the same level. Due to the complex actual situation of the site and the influence of the technical conditions of the monitoring technique, the time interval of monitoring events can only be controlled within a long-time interval which will also have a certain impact on the prediction of results.

### 4.2. Limitations and Prospects

It is clear that a method to build a risk field model based on in-situ microseismic monitoring data can be proposed. However, there are three points of view that need to be noted inorder to continuously improve and develop this study.

a. Effect of microseismic monitoring equipment performance and positioning accuracy.

As previously mentioned, this study can provide a method to assess and calculate the 3D risk value. However, the extremely complex field conditions and susceptible interference from noise signals will have a great impact on the positioning results, which is the main reason of errors for the in-situ data analysis. Therefore, this study proposes a risk field model under ideal conditions. If this study can be combined with the in-situ actual situation, the spatial distribution pattern of risk values may be reflected more realistically. With the increasing improvement of microseismic monitoring technology and the continuous optimization of positioning methods, we can continuously improve the accuracy of the collected data. Thus, further improvement and development could be made to optimize risk field theoretical model.

b. Calculation of probability values.

Since the focus of this study is concentrated on the analysis of individual microseismic events, the Copula function is capable of analyzing the probability distribution of the discrete data selected to calculate the probability distribution values. In this study, the Copula function of the two-dimensional function was chosen for the solution of the probability values. We can select multi-dimensional Copula function to optimize the model in future researches. Also the probability values calculated in this study are the probabilities of the distributions obtained from discrete data. Therefore, an in-depth study can be carried out to calculate probability values to make them more relevant in future research.

c. Dynamic risk field model.

The complex topographic conditions at the site create a number of difficulties in providing more accurate attenuation equations. The wave velocity attenuation can be analyzed in the future by monitoring a large number of microseismic field monitoring data over a certain continuous time period. If more accurate wave velocity attenuation is known, then its specific distribution over a certain distance and time can also be known. In this case the analytical model presented in Section 2.4 will be more accurate. Also, if the sampling interval of the device is small enough, the amount of data collected in a certain time will be significantly increased. The analysis of a large amount of continuous monitoring data within a certain time will be the focus of future research on dynamic risk field theory.

## 5. Conclusions

In this study, a microseismic monitoring data based risk field model was proposed to evaluate the regional risk of rock instability in a certain area. The rock instability risk levels of the assessed area can be confirmed by 3D risk assessment. In conclusion, the variable relationship between risk and coordinates can be established and characterized. According to the verification of validity and availability of the proposed method, accurate risk assessment results of rock instability can be obtained based on the 3D risk function. Therefore, the proposed risk field can be effectively applied to the quantitative risk assessment of rock instability and can provide a new perspective for the development of risk assessment of rock instability.

## Figures and Tables

**Table 1:** Weight distribution table for each indicator.

Parameter	Index	Weight
PGV	ϑ_1_	0.245
PGA	ϑ_2_	0.299
Energy flux	ϑ_3_	0.455

**Table 2:** The calculation results of severity of t0: 201401040322.

Sensor Serial Number	S_1_	S_2_	S_3_	Severity
1	8.10 × 10^−8^	5.04 × 10^−5^	2.92 × 10^−9^	1.51 × 10^−5^
2	8.27 × 10^−8^	8.26 × 10^−6^	3.20 × 10^−9^	2.49 × 10^−6^
3	4.90 × 10^−7^	1.60 × 10^−4^	7.88 × 10^−8^	4.79 × 10^−5^
6	3.45 × 10^−5^	4.60 × 10^−3^	3.21 × 10^−4^	1.53 × 10^−3^
7	2.46 × 10^−6^	9.47 × 10^−4^	3.26 × 10^−6^	2.85 × 10^−4^
8	2.37 × 10^−6^	3.76 × 10^−4^	8.45 × 10^−7^	1.13 × 10^−4^
9	1.50 × 10^−5^	3.58 × 10^−3^	1.83 × 10^−5^	1.08 × 10^−3^
11	2.07 × 10^−4^	4.11 × 10^−4^	2.80 × 10^−2^	1.29 × 10^−2^
12	1.34 × 10^−5^	4.08 × 10^−3^	1.09 × 10^−4^	1.27 × 10^−3^
13	7.56 × 10^−6^	1.22 × 10^−3^	1.29 × 10^−5^	3.72 × 10^−4^
16	5.16 × 10^−7^	2.32 × 10^−4^	2.40 × 10^−7^	6.97 × 10^−5^
17	4.65 × 10^−7^	1.23 × 10^−4^	5.63 × 10^−8^	3.71 × 10^−5^
18	4.17 × 10^−7^	1.09 × 10^−4^	4.38 × 10^−8^	3.26 × 10^−5^
25	4.38 × 10^−5^	3.04 × 10^−2^	1.25 × 10^−3^	9.66 × 10^−3^
26	1.65 × 10^−5^	8.60 × 10^−3^	9.55 × 10^−5^	2.62 × 10^−3^
27	1.77 × 10^−7^	1.24 × 10^−4^	1.67 × 10^−7^	3.72 × 10^−5^
28	2.32 × 10^−7^	1.34 × 10^−4^	3.44 × 10^−8^	4.01 × 10^−5^

**Table 3:** Calculation results of AIC and BIC.

Copula Function	AIC	BIC
Gaussian	−18.4695	−17.6363
t-Copula	−20.3901	−19.5569
Gumbel	−13.6901	−12.8569
Clayton	−34.407	−33.5738
Frank	−22.888	−22.0547

**Table 4:** Risk value calculation results.

Sensor Number	Severity	Probability	Risk
1	1.51 × 10^−5^	0.18	2.65 × 10^−6^
2	2.49 × 10^−6^	0.16	4.01 × 10^−7^
3	4.79 × 10^−5^	0.24	1.16 × 10^−5^
6	1.53 × 10^−3^	0.79	1.21 × 10^−3^
7	2.85 × 10^−4^	0.55	1.55 × 10^−4^
8	1.13 × 10^−4^	0.33	3.78 × 10^−5^
9	1.08 × 10^−3^	0.61	6.61 × 10^−4^
11	1.29 × 10^−2^	0.32	4.17 × 10^−3^
12	1.27 × 10^−3^	0.72	9.11 × 10^−4^
13	3.72 × 10^−4^	0.58	2.17 × 10^−4^
16	6.97 × 10^−5^	0.31	2.20 × 10^−5^
17	3.71 × 10^−5^	0.21	7.65 × 10^−6^
18	3.26 × 10^−5^	0.20	6.45 × 10^−6^
25	9.66 × 10^−3^	0.90	8.67 × 10^−3^
26	2.62 × 10^−3^	0.78	2.03 × 10^−3^
27	3.72 × 10^−5^	0.42	1.56 × 10^−5^
28	4.01 × 10^−5^	0.22	8.75 × 10^−6^

**Table 5:** Comparison and summary of inspection results.

Data Comparison Group	Sensor Serial Number	Risk Value without Disturbance	Risk Value with Disturbance	Ratio of Risk Value
Comparison of group data at time T1 and t1	12	2.11 × 10^−6^	3.14 × 10^−5^	14.90
	13	1.60 × 10^−6^	8.02 × 10^−5^	50.05
	6	3.40 × 10^−6^	6.24 × 10^−3^	1838.85
	18	1.45 × 10^−6^	8.67 × 10^−7^	0.60
	27	2.97 × 10^−7^	2.58 × 10^−6^	8.71
Comparison of group data at time T2 and t2	22	3.40 × 10^−8^	1.04 × 10^−7^	3.04
	21	2.75 × 10^−7^	1.10 × 10^−5^	39.95
	2	7.32 × 10^−7^	2.47 × 10^−6^	3.37
	20	1.57 × 10^−6^	5.08 × 10^−5^	32.40
	18	3.01 × 10^−5^	2.03 × 10^−5^	0.68
	19	6.27 × 10^−6^	4.73 × 10^−5^	7.54
Comparison of group data at time T3 and t3	18	7.98 × 10^−7^	4.30 × 10^−5^	53.85
	3	5.13 × 10^−6^	1.02 × 10^−4^	20.00
	17	6.39 × 10^−6^	1.52 × 10^−5^	2.39
	16	9.06 × 10^−7^	2.00 × 10^−6^	2.20

## Data Availability

Not applicable.
